# Psychometric properties of the Short Form-8 Health Survey (SF-8) among diabetes and non-diabetes Iranian older people

**DOI:** 10.34172/hpp.2021.43

**Published:** 2021-08-18

**Authors:** Shamsedin Namjoo, Masoud Mirzaei, Mahshid Foroughan, Gholamreza Ghaedamini Harouni

**Affiliations:** ^1^Department of Aging, University of Social Welfare and Rehabilitation Sciences, Tehran, Iran; ^2^Yazd Cardiovascular Research Center, Shahid Sadoughi University of Medical Sciences, Yazd, Iran; ^3^Iranian Research Center on Aging, Department of Aging, University of Social Welfare and Rehabilitation Sciences, Tehran, Iran; ^4^Social Welfare Management Research Center, University of Social Welfare and Rehabilitation Sciences, Tehran, Iran

**Keywords:** Quality of life, Eight-Item Short-Form Health Survey (SF-8), Health, Psychometrics, Iran

## Abstract

**Background:** The current study aimed to evaluate the psychometric properties of the Persian version of the 8-item Short-Form Health Survey (SF-8). For this purpose, we examined a large sample of the older adult in two different groups with and without diabetes using the YazdHealth Study (YaHS) data.

**Methods:** Using a two-stage cluster random sampling method, 1901 older adults were recruited, according to the World Health Organization (WHO) STEPwise approach to surveillance(STEPS) guidelines. To test the scale’s reliability, the internal consistency and test-retest methods were applied. The convergent validity of the entire questionnaire was evaluated by the average variance extracted (AVE) and composite reliability (CR) for each subscale. An independent samples t-test was used to assess the demographic differences between the study groups.

**Results:** The Cronbach’s alpha coefficient for the subscales of SF-8 were measured to range between 0.85 and 0.79 (physical & mental health). The test-retest reliability coefficient of the physical component summary (PCS) and (0.97) and mental component summary (MCS) (0.98)indicated the appropriate reliability of the SF-8. The CFA-concerned results indicated that the the2‐factor model presented a good fit to the data for the explored diabetes and non-diabetes groups, as well as the total research participants [goodness of fit index (GFI)=0.99, comparative fit index (CFI)=0.992, normed fit index (NFI)=0.99, incremental fit index (IFI)=0.992, root mean square error of approximation (RMSEA)=0.056]. Values >0.5 and >0.7 for AVE and CR indicated the evidence of the convergent validity of the SF-8.

**Conclusion:** The present study was the first attempt to confirm the traditional 2-factor structure of SF-8 among a large sample of Iranian older individuals. The obtained results suggested that the Persian version of the SF-8 is a reliable and valid tool for measuring health-related quality of life (HRQoL) among Iranian older adults (including the older adult with & without diabetes).

## Introduction

Health-related quality of life (HRQoL) is an aspect of quality of life (QoL). Besides, the HRQoL is a reliable index for assessing the health status of individuals.^1^ QoL is defined as the perception of individuals about their position in life, concerning the cultural context and the value system in which they live, and respecting their goals, expectations, standards, and concerns.^2^ The QoL is a concept that helps healthcare researchers to explore the health status of different populations and their associated factors. Furthermore, it is beneficial in evaluating the effects of health intervention programs.^3^

Age is a major factor affecting the HRQoL^4^; thus, it is essential to assess HRQoL in aging populations. Currently, population aging is a global phenomenon,^5^ encompassing developed and developing countries. Iran, as a developing country, is also experiencing a similar population alternation. Moreover, Iran’s aged population will be two folds higher in the next 3 decades (approximately 25% of the total population).^6^ Assessing HRQoL requires developing valid and reliable instruments to reflect a precise picture of the community and healthcare system. A valid tool assists healthcare policymakers and providers to promote their functions in delivering healthcare services to the target populations.^7,8^

The most recognized tools to evaluate the QoL consist of the 36-Item Short-Form Health Survey (SF-36) and the World Health Organization Quality of Life Brief Version (WHOQOL-BREF).^9^ Of the briefest general questionnaires of QoL, the 8-item Short-Form Health Survey (SF-8) (an abbreviated version of the original SF-36) is among the most widely used QoL assessment scales, worldwide. Our 5 reasons for choosing this questionnaire to study were as follows: it is easily applicable; its completion requires a short time; it is age-friendly,^10,11^ also it can be implemented regardless of age, illness, or treatment, and the level of education.^12^ As per the general consensus, adapting the original high-quality questionnaires and validating them in a culturally different population is more practical and economical than designing and validating a new one.^13^ Additionally, some questionnaires have been designed in Iran to assess the HRQoL over the past decades; most of them are not popular due to their specific nature (assessing QoL exclusively in individuals with diabetes or coronary artery disease), too many items, or the long time taken to be completed.^14,15^

The psychometric properties of the SF-8 have been examined in various studies. The obtained Cronbach’s alpha coefficient (0.85) in a study in China suggested that the SF-8 has appropriate reliability. The same study also indicated that a 3-factor model (physical, mental, & overall health) better fits the data than the conventional 2-factor pattern.^10^ Onagbiye et al^11^ assessed the validity and reliability of the Setswana Short-Form Health-Related Quality of Life Survey (SF-8) in adults. The Setswana SF-8 presented good concurrent validity with the Spearman’s correlation coefficient (*ρ*) of 0.72 for physical component summary (PCS) to 0.91 for social functioning. The Cronbach’s alpha coefficients for the first and second measurements were computed as 0.87 and 0.87, respectively. Moreover, Tomás et al^16^ indicated that the traditional 2-factor model of the Spanish version of the SF-8 demonstrated a good fit to the data.

According to the literature, no study has examined the psychometric properties of the SF-8 in Iran, especially among the older adult. Thus, this study aimed to evaluate the psychometric properties of the Persian version of the SF-8. The main research purpose was to explore whether SF-8 is a proper instrument for assessing HRQoL among the Iranian older adult.

All study subjects were provided a written informed consent form to participate in this research. Furthermore, they received explanations about the confidentiality of their data, identity, as well as the right to withdraw from the study at any stage. We observed the Yazd Health Study (YaHS) experiment protocol for involving human data as the guidelines of national human ethics (available at https://ethics.research.ac.ir/) as well as the Helsinki Declaration of 1964, 2000 revision.

## Materials and Methods

### Study design, participants and procedure 

We used the data obtained from the recruitment phase of the YaHS. The present study included data gathered from 1901 older adult (age: >60 years) resident of Yazd. The research participants were divided into two groups (696 subjects with diabetes & 1205 individuals without diabetes). Initially, 200 clusters were randomly selected from 3 areas of Yazd City, Iran, by multistage stratified sampling method and details of the methodology were published elsewhere^17^. The demographic questionnaire and the Persian version of SF-8 were used for data collection.

### Scale preparation steps

***Adaptation procedure***

Several steps were taken to translate the SF-8 instrument based on the international guidelines to assure the accuracy of the translation procedure, as follows: (1) forward translation: two bilingual native Iranians with a background in social sciences and gerontology independently translated the SF-8 from English into Persian. (2) Both translators and a project manager compared the translated versions and discussed unifying the two translated versions. (3) Backward translation: two professional English language translators back-translated this Persian version into English.

The translators of the second step were blinded to the original English version of the SF-8. This measure was taken to identify conceptual inconsistencies between the translated and original versions of the questionnaire. (4) Each item and the entire questionnaire were reviewed by the research team; eventually, the questionnaire was approved with the consensus of all members. (5) The SF-8 was implemented on 50 older adult to understand how they interpret the items of the questionnaire. (6) In the last step, all the necessary modifications were applied based on the suggestions obtained from the initial stages as well as the pilot study on the final version of the questionnaire. Finally, the SF-8 was implemented on the 1901 older adult.

### Short Form Health-Related Quality of Life (SF-8)

The SF-8 is a short version of the original 36-item Short-Form Health Survey (SF-36). It has 8 domains, including general health, physical functioning, role limitations due to physical problems, bodily pain, vitality, social functioning, mental health, and role limitations due to emotional problems. It is a generic multipurpose short-form quality of life instrument developed by the RAND Corporation and the Medical Outcomes Study (MOS) in the 1980s,^18^ with two PCS and mental component summary (MCS) dimensions.

### Ceiling and floor effects

The range of the measured scores was examined by computing ceiling (the maximum possible score) and floor (the minimum possible score) effects. Ceiling and floor effects are considered to be present if >20% of the respondents report the lowest or highest possible total scores, respectively.^19^

The acceptability of the SF-8 items was measured by calculating missing values, as well as ceiling and floor effects. There were minimal missing items (<5%). No floor and ceiling effects were identified for all the SF-8 subscales.

### Content validity

The prepared questionnaire was provided to an expert panel (7 members) of different disciplines, including gerontology, social welfare, epidemiology, and health education for reviewing its content validity. They were requested to comment on the relevancy, clarity, and simplicity of the items. To analyze the data respecting the content validity, two indicators, including content validity ratio (CVR) and content validity index (CVI) were used. The CVI of ≥0.79 and CVR of ≥0.75 were considered acceptable for each item.^20^

### The assessment of internal consistency, reliability, and stability

To determine the questionnaire’s internal consistency and reliability, Cronbach’s alpha coefficient and test-retest method were employed. Furthermore, the Intra-class correlation coefficient (ICC) of the scale was calculated. Additionally, the questionnaire was tested with a 14-day interval through the completion of the scale by 30 participants. Minimum Cronbach’s alpha coefficient of 0.7 and ICC of 0.6 was considered as acceptable.^19^

### The construct validity of the questionnaire

Discriminant validity, convergent validity, and confirmatory factor analysis (CFA) approaches were applied to determine the construct validity of the tool.

### Confirmatory Factor analysis

The CFA technique was used to examine the construct validity of the SF-8. In addition to presenting a significant factor loading of ≥0.40, the comparative fit index (CFI≥0.90), the Incremental fit index (IFI ≥ 0.90), the root mean square error of estimation (RMSEA ≤ 0.08), and the Goodness of Fit Index (GFI ≥ 0.90) were used to assess the measurement model fit to the data.^21^

### Discriminant validity

Previous studies revealed that the QoL of individuals with diabetes is poor, compared to their non-diabetics counterparts.^22^ Therefore, Known Group Comparison was assessed by comparing the SF-8 scores between the study groups by the Student’s Samples t-tests and Independent Samples *t* test at *P* < 0.05. Besides, the square root of average variance extracted (AVE) was used for evaluating the discriminant validity of the entire questionnaire.

### Convergent validity:

AVE and composite reliability (CR) were computed using the completely standardized loading extracted from the CFA. Values >0.5 and >0.7 for AVE and CR indicated the evidence of the convergent validity of the SF-8, respectively. We used the following formula for calculating

the AVE and CR.

$$AVE=\frac{\Sigma \lambda^{2}}{n}$$

$$CR=\frac{{\left(\Sigma \lambda \right)}^{2}}{{\left(\Sigma \lambda \right)}^{2}+\;\Sigma^{\delta }}$$

λ_i_ = completely standardized loading for the 

*i*th indicator, δ_i_ = variance of the error term for the *i*th indicator,

n = number of indicators

### External validity

The only formal approach to establishing the external validity of a scale is to repeat the study in that specific target population.^23^ Therefore, the SF-8 was used in 8000 subjects, aged 20-70 years for assessing its external validity.

### The normality of the data

Skewness and kurtosis were used for assessing the normality of the obtained data. Skewness indices ranged from −0.09 to 1.002 and kurtosis indices ranged from −0.02 to 0.4, indicated the normality of the collected data.

### Data analysis

The achieved data were analyzed in SPSS version 18 (IBM Corp. ARMONK, USA) and AMOS version 18 (IBM SPSS) at *P* ≤ 0.05.

## Results

This study included 1901(1205 non-diabetes & 696 diabetes) respondents; of whom, 963 (50.7%) were males. In total, 61.1% of the study subjects reported an educational level of primary school and below, and 87.3% of them were married (Table 1).

**Table 1:** The socio-demographic characteristics of the respondents (N = 1901)

**Variables**	**Group**	**n**	**%**
Gender	Female	938	49.3
	Male	963	50.7
Educational level	Primary school and less	1165	61.3
	Secondary	416	21.9
	Diploma	249	13.1
	Masters and Ph.D.	71	3.7
Marital status	Single	20	1.1
	Married	1660	87.3
	Widow/divorced	221	11.6

An expert panel consisting of academic staff members of the University of Social Welfare and Rehabilitation Sciences and Isfahan University of Medical Sciences assessed the CVI and CVR of the explored tool. The CVI and CVR values were calculated based on the respondents’ answers to the items concerning the relevancy of the questionnaire’s items. A CVI of 0.97 and CVR of 0.98 was considered acceptable for the entire questionnaire (Table 2).

**Table 2:** Psychometric properties of the SF–8

**Item**	**Factor loadings (Beta)**	**Item-total correlation**	**Mean**	**CVR**	**CVI**	**Internal consistency**	**Test-retest reliability**	**AVE**	**CR**
VT	0.565*	0.55	51.8	0.85	0.92	0.79	0.98	0.90	0.88
SF	0.760*	0.68	36.0	1	1				
RE	0.658*	0.58	37.3	1	0.96				
MH	0.825*	0.73	36.8	1	0.98				
GH	0.656*	0.63	41.8	1	1	0.85	0.97	0.82	0.86
PF	0.840*	0.72	43.1	1	0.94				
RP	0.881*	0.76	41.1	1	0.98				
BP	0.701*	0.66	48.7	1	0.98				

GH, general health; PF, physical functioning; RP, role limitations due to physical problems; BP, bodily pain; VT, vitality; SF, social functioning; MH, mental health, RE, role limitations due to emotional problems; CVR, content validity ratio; CVI, content validity index; AVE, average variance extracted; CR, composite reliability. *P value <0.001.

The internal consistency of the scale’s total score (Cronbach’s alpha coefficients, ranging from 0.87 to 0.80 for PCS & MCS subscales in the older adult with diabetes and 0.83-0.78 for the same subscales in the non-diabetes group) and for the entire questionnaire (ranging from 0.84 to 0.79 for PCS & MCS subscales) was considered acceptable. To measure the test-retest reliability of the Persian version of the SF-8, 20 older people were recruited to complete the SF-8 twice with a 14-16-day interval. The test-retest correlation coefficients of the PCS and MCS subscales of the SF-8 were computed as 0.97 and 0.98, respectively (Table 2).

Values >0.5 and >0.7 for AVE and CR indicate the evidence of the convergent validity of the SF–8, respectively. The AVE (0.9 to 0.82 for MCS & PCS, respectively) and CR (0.82 to 0.88 for PCS & MCS, respectively) indices established evidence of the convergent validity of the SF–8 (Table 2).

The CFA data demonstrated a good fitness for two factors of SF-8 in the study groups (Table 2). The GFIs were almost the same in both research groups, indicating the applicability of the questionnaire in different groups, as well as the general older adult population; the items were examined in terms of factor load and in the research groups (Table 3).

**Table 3:** Goodness-of-fit indices of models for two groups and the total participants

**CI**	**RMSEA**	**IFI**	**NFI**	**CFI**	**GFI**	***P*** ** value**	**χ** ^ 2 ^ **/df**	**df**	**χ** ^ 2 ^	**Groups**
0.04-0.07	0.056	0.992	0.99	0.992	0.99	0.001	4.4	10	44.46	Total

*Note*. GFI, goodness of fit index [good fit: ≥ 0.9]; CFI, comparative fit index [good fit: ≥ 0.9]; IFI, incremental fit index [good fit: ≥0.9]; NFI, normative fit index [good fit: ≥0.9]; RMSEA, root mean square error of approximation [good fit: <0.08; fair fit: 0.08–0.10]; 95% CI, Confidence Interval of RMSEA.

Evaluating the psychometric properties of the questionnaire in a population of 8000 individuals, aged 20 to 70 years presented a good fitness to the data (GFI = 0.98, CFI = 0.98, IFI = 0.98, & RMSEA = 0.06) and acceptable Cronbach’s alpha coefficient for the two factors (PCS=0.85 & MCS = 0.80) of the SF–8.

According to the obtained results, there was no relationship between MCS and PCS and gender and the literacy status in the explored older adult with diabetes (*P* > 0.05). In the non-diabetes older adult group, there was a significant relationship between gender and PCS (*P* = 0.001) and MCS (*P* = 0.001); however, no significant correlation was detected between any aspects of HRQoL and educational level in the study participants.

Comparing the study groups to determine the relevant discriminant validity highlighted that the mean difference of PCS in the older adult group with diabetes was 1.63 (*P* = 0.001, CI: 1.32–1.93), and in non-diabetes individuals was 1.47 (*P* = 0.001, CI: 1.2–1.7). The results showed a statistically significant difference and also based on the result from the Square root of AVE, it can be concluded that the tool has an appropriate discriminant validity (Table 4).

**Table 4:** Discriminate validity of SF-8 by using known groups

**Factor**	**Diabetes older adult(n = 696)** **Mean ± SD**	**Non-diabetes older adult(n = 1205)** **Mean ± SD**	***P*** ** value**	**Mean difference**	**95% Confidence Interval**	**Latent correlation**	**Square root of AVE**
PCS	10.15 ± 3.1	8.52 ± 3.35	0.001	1.63	1.32–1.93	PSC<--- HRQOL 0.44	0.77
MCS	8.97 ± 2.7	7.49 ± 2.9	0.001	1.47	1.2–1.7	MSC<---HRQOL 0.49	0.70

## Discussion

This study assessed the psychometric properties of the SF–8. The SF-8 can be used as a short appropriate tool for measuring HRQoL among the Iranian older adult. To the best of our knowledge, this study was the first attempt concerning this questionnaire, i.e., conducted in a large sample size with different groups.

The present research results indicated the desired reliability of the SF-8 by high levels of internal consistency. Moreover, this finding was consistent with those of Lang et al^10^ (Cronbach’s alpha coefficient: 0.82) and Onagbiye et al^11^ (Cronbach’s alpha coefficient: 0.87).

**Figure 1:**
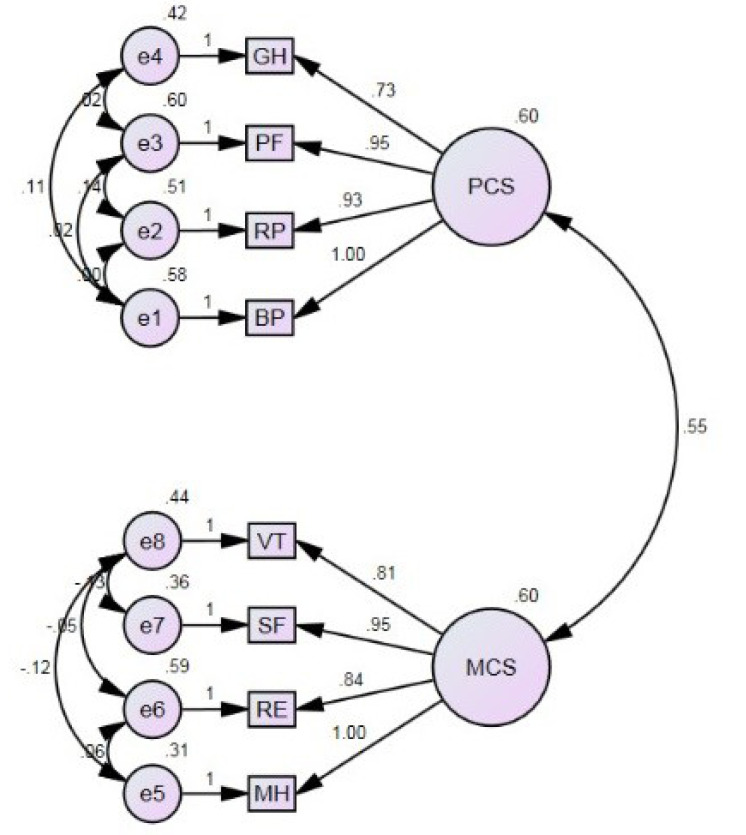
Confirmatory factor analysis of the Short Form-8 Health Survey (SF-8)

The results of the confirmatory factor analysis in the examined older adult with and without diabetes revealed that the presumed two-dimensional model (Figure 1) of the SF–8 provided an acceptable similar structural validity, i.e., consistent with the results of previous studies.^10,24^ Other investigations suggested that a 3-factor model (physical, mental, and overall health) better fit the data than the traditional 2-factor model.^10^ The discrepancies between our study and prior research can be explained by different sample sizes (in the present study, the sample size equaled 1901 subjects, while in the previous study, the sample size consisted of 10885 individuals),^10^ target groups, or the heterogeneity of participants in different age groups (the present study was performed on younger older population and the other studies examined the general population).^10^

Since, 8000 people were selected from the same first community (1901 older adult), and by the same sampling method people to generalization of the results to other populations and prevent possible bias, based on the results, it can be claimed that SF–8 had appropriate external validity as well as goodness of fit index.

Based on our results, the calculated convergent validity, discriminant validity, and external validity were acceptable for the SF-8, i.e., not evaluated in the previous studies.

The results of the current study, similar to Lee and Shinkai’s^25^ and Zare et al^26^ research, signified no significant relationship between the dimensions of SF-8 and gender and level of education in the older adult with diabetes; however, this result was in contrast with those of other studies.^25-27^ A significant relationship was observed between gender and QoL among non-diabetes older individuals, i.e., consistent with some previous studies.^24^ The gender-wise differences in the overall study results can be attributed to culture, environment, and sample size. A large body of literature indicated that the QoL was higher in males, compared to female.^26-27^ Our findings highlighted the ineffectiveness of the literacy level on the scores of the questionnaire, i.e., in line with those of previous investigations.^12,28,29^

### Strengths

The present study was the first attempt to evaluate the psychometric properties of the SF-8 in the Iranian older adult. Using a large sample size can be considered among the strengths of the present study.Another strengths of this study was examine the external validity, which was not addressed in previous studies.

## Conclusion

Since, the results of the present study showed that the SF-8 scale has good reliability and validity, and also based on the study of external validity and the ability to generalization, it can be claimed that the present scale, regardless of literacy, it will be useful in the Iranian older adult. Therefore, this scale can be used in various studies, including epidemiological studies, clinical studies, and efficacy assessment of health-related interventions, due to its easy to use and low number of items.

## Funding

This article is part of a Ph.D. thesis in gerontology, which was supported and approved by the University of Social Welfare and Rehabilitation Sciences, Tehran, Iran. The funders had no role in study design, data collection, and analysis, decision to publish, or in the preparation of the manuscript.

## Competing interests

The authors reported no potential conflict of interest.

## Ethics approval

This study was approved by the Ethics Committee of the University of Social Welfare and Rehabilitation Sciences (IR.USWR.REC.1398.006) and Shahid Sadoughi University of Medical Sciences (No.17/1/73941). All subjects provided written informed consent after explaining the confidentiality of their responses.

## Authors’ contributions

SN, MF, MM and GGH designed the study. MM collected survey data. SN, MF, MM and GGH analyzed and presented the statistical results. SN, MF, MM and GGH were major contributors in writing the manuscript. SN, MF and MM edited the manuscript. All authors read and approved the final manuscript.
